# Supplementary material for: Evaluating language policy implementation in South African higher education - three decades of progress and challenges: A scoping review protocol
Source: PLoS One. 2025 Apr 25;20(4):e0322322. doi: 10.1371/journal.pone.0322322 (PMC12027021; doi:10.1371/journal.pone.0322322)
Supplement: Attachment — Submitted filename: Reviewers report PLOS ONE.docx [file pone.0322322.s001.docx]

**Reviewer’s report on Manuscript Number: PONE-D-24-54236**

The authors attempted a scoping review to evaluate the current state of language policy implementation in South African public higher education institutions

**General overview of the protocol**

- The protocol is presented in an intelligible fashion and is written in standard English.
- The protocol meets all applicable standards for research integrity.
- The protocol adheres to appropriate reporting guidelines.
- The protocol meets scientific soundness and does not lack novelty.

1. Recommendation

(Answer options: Accept, *Accept with Minor Revision*, Major Revision, Reject)

2. Is the manuscript technically sound, and do the data support the conclusions?

(Answer options: *Yes,* No, Partly)

3. Has the statistical analysis been performed appropriately and rigorously?

(Answer options: Yes, No, I don’t know, *N/A*)
4. Does the manuscript adhere to the PLOS Data Policy? Additional details can be found at http://journals.plos.org/plosone/s/materials-and-software-sharing. (Answer options: Yes, *No*)
5. Is the manuscript presented in an intelligible fashion and written in standard English? (Answer options: *Yes*, No)
6. Review Comments to the Author (minimum 200 characters)

- Abstract: Please add the dates of coverage to the abstract ==methods section.
- The abstract exceeded ***300*** words. So, please revise it to follow the structured abstract recommended words in PLOS One Open’s Instructions for Authors for study submission. See: https://journals.plos.org/plosone/s/submission-guidelines#:~:text=The%20Abstract%20comes%20after%20the,objective(s)%20of%20the%20study
- Line 11: authors wrote specifically, *it seeks to:* So, they ended the preamble with a “:” and they went on to use a “,” to separate each specific objective. I suggest if a “:” is used, then a “;” is ideal to separate each point then end with a period. E.g., 1) map the integration of multilingual policies into institutional teaching, research, and administrative practices; (2) identify persistent barriers to effective policy implementation.
- Please, in the text, cite the reference number in square brackets e.g., “[19]” not in parenthesis “(19)”. Therefore, do thorough revision to effect this.
- Please, separate where two different articles are cited with “,” e.g., [10], [11] not (10) (11) remember it should be bracket square and not parenthesis.
- Wherever a “:” is used to indicate a list of points, please separate each point with a “;” and end with a period.
- Why is the search restricted to 1994 to present (three decades)? Please justify in the methods section.
- I suggest you delete the conclusion section for it is not required for protocol articles.
- Line 37: (3), argue for comprehensive language policies spanning all educational levels, including higher education, to enhance accessibility and inclusivity. I suggest reviewers can mention the author’s last name without the year of publication. E.g., Amoah [3], argues that for comprehensive language policies spanning all educational levels, including higher education, to enhance accessibility and inclusivity.

7. PLOS authors have the option to publish the peer review history of their article (https://journals.plos.org/plosone/s/editorial-and-peer-review-process#loc-peer-review-history). If published, this will include your full peer review and any attached files. Do you want your identity to be public for this peer review? (Answer options: Yes, *No*)
7a. If you answered "Yes" above, that you would like your identity to be public, please provide your full name, as it should appear on the published peer review. Please do not sign the review on behalf of another person.

Confidential to Editor:
1. Do you have any potentially competing interests? If none, type "None." Our policy on competing interests can be found at <http://journals.plos.org/plosone/s/competing-interests>.

Answer: *None*
2. Did you receive any assistance in preparing this review (e.g. from a post-doc or graduate student)? If yes, please provide their name and title. If you have chosen to reveal your identity to the authors in question 7 above, please include the name of the person who assisted you in the response to question 7 as well. (optional)

Answer: *No*
3. Do you want to get recognition for this review on Web of Science (https://clarivate.com/products/scientific-and-academic-research/research-discovery-and-workflow-solutions/researcher-profiles/)? If you opt in, your Web of Science profile will automatically be updated to show a verified record of this review in full compliance with the journal's review policy. If you don't have a Web of Science profile, you will need to first create a free account. (Answer options: *Yes*, No)
