# Supplementary material for: Evaluating language policy implementation in South African higher education - three decades of progress and challenges: A scoping review protocol
Source: PLoS One. 2025 Apr 25;20(4):e0322322. doi: 10.1371/journal.pone.0322322 (PMC12027021; doi:10.1371/journal.pone.0322322)
Supplement: Attachment — Submitted filename: Search-Suggestions.docx [file pone.0322322.s002.docx]

| Concept | Strategy |
| --- | --- |
| #1 Language policy | "language policy" OR "language policies" OR "language plan*" |
| #2 South African Higher Education | ((university OR universities OR college OR colleges OR "higher education" OR "higher ed") AND "South Africa" OR South African")) OR (LIST ALL PUBLIC UNIVERISITIES THAT WOULD BE INCLUDED) |
| #3 Languages | Multilingual OR "multi lingual" OR "multi-lingual" OR bilingual OR bi-lingual OR "bi lingual" OR trilingual OR tri-lingual OR "tri-lingual" OR "indigenous language" OR "indigenous languages" OR "native language" OR "native languages" OR LIST ALL LANGUAGES THAT WOULD BE INCLUDED |
| #4 | #1 AND #2 AND #3 |
