# Supplementary material for: Evaluating language policy implementation in South African higher education - three decades of progress and challenges: A scoping review protocol
Source: PLoS One. 2025 Apr 25;20(4):e0322322. doi: 10.1371/journal.pone.0322322 (PMC12027021; doi:10.1371/journal.pone.0322322)
Supplement: Attachment — Submitted filename: Rebutabble Table.docx [file pone.0322322.s004.docx]

We would like to sincerely thank all the reviewers for their thoughtful and constructive comments on our protocol. Your feedback has significantly contributed to enhancing the quality of our work. We have addressed all comments to the best of our ability and appreciate the time you took to review the protocol thoroughly. Below is a rebuttal table outlining each reviewer’s comment and our corresponding response, along with the page and line numbers where changes have been made.

| **Reviewers** | **Comments** | **Responses** | **Pages number** |
| --- | --- | --- | --- |
|  | Finally, the discussion should acknowledge the role of English as the dominant language of scientific communication, particularly in graduate and postgraduate education, where publishing requirements may present challenges for non-English speakers. | Thank you for your comment. We agree that English plays a dominant role in scientific communication, especially in graduate and postgraduate education, where publishing requirements can pose challenges for non-English speakers. We have adjusted the discussion to explicitly acknowledge this point, while also emphasizing the importance of balancing the use of English with the promotion of linguistic diversity to support inclusivity in academia. | Page 16 - 17, line 278 – 294. |
| 1 | Abstract: Please add the dates of coverage to the abstract ==methods section. | Thank you for your comment. We have now incorporated the coverage dates (1994 to present) in the Methods section of the abstract to enhance clarity. | Page 2, line 17. |
|  | The abstract exceeded 300 words. | Thank you for this comment, the abstract words have been adjusted to 292 words | Page 2 - 3, line 2 – 27. |
|  | Line 11: authors wrote specifically, it seeks to: So, they ended the preamble with a “:” and they went on to use a “,” to separate each specific objective. I suggest if a “:” is used, then a “;” is ideal to separate each point then end with a period. E.g., 1) map the integration of multilingual policies into institutional teaching, research, and administrative practices; (2) identify persistent barriers to effective policy implementation. | Thank you for your suggestion. We have revised the sentence to use semicolons (;) to separate each specific objective and ensured the final point ends with a period, as recommended. | Page 2, line 9 – 13  Page 5, line 88 -90. |
|  | Please, in the text, cite the reference number in square brackets e.g., “[19]” not in parenthesis “(19)”. Therefore, do thorough revision to effect this. | Thank you for your comment. All the in-text references are in square brackets throughout the protocol. |  |
|  | Please, separate where two different articles are cited with “,” e.g., [10], [11] not (10) (11) remember it should be bracket square and not parenthesis. | Thank you for your comment. We have revised the citations to ensure that multiple references are separated by commas and enclosed in square brackets as per the required formatting style. | Page 3, line 42 page 4, line 54. |
|  | Wherever a “:” is used to indicate a list of points, please separate each point with a “;” and end with a period. | Thank you for your comment. We have adjusted the text to separate each point with a semicolon and to end each list with a period. | Page 2, line 9 – 14  Page 5, line 88 – 90. |
|  | Why is the search restricted to 1994 to present (three decades)? Please justify in the methods section. | Thank you for your comment. We have now included a justification for restricting the search to studies published from 1994 to the present in the Methods section under the Search Strategy. This timeframe aligns with the post-apartheid era in South Africa, a period marked by significant language policy reforms, including the 1996 Constitution and subsequent higher education language policies. Restricting the review to this period ensures that it captures literature reflecting these policy changes and their impact on multilingualism in higher education over the past three decades. | Page 7, line 107 – 112. |
|  | I suggest you delete the conclusion section for it is not required for protocol articles. | Thank you for your comment. We have deleted the conclusion. |  |
|  | Line 37: (3), argue for comprehensive language policies spanning all educational levels, including higher education, to enhance accessibility and inclusivity. I suggest reviewers can mention the author’s last name without the year of publication. E.g., Amoah [3], argues that for comprehensive language policies spanning all educational levels, including higher education, to enhance accessibility and inclusivity. | Thank you for your comment. We have revised the sentence to mention the author’s last name without the year of publication, as recommended. The revised sentence reads: (Xulu-Gama & Hadebe), argue for comprehensive language policies spanning all educational levels, including higher education, to enhance accessibility and inclusivity. | Page 3 line 36 – 37. |
| 2 | This was very clear, and even as a non-expert in research, I could follow the process. I find this proposal fascinating as a professor of education, because in the US, we often find working with 2-3 languages challenging! Your study is a good "state of the union" analysis to see how reforms are working and I look forward to reading what you discover. I only discovered a small error on page 3 line 37, where I think part of a sentence is missing. | Thank you for your comment. We will be glad to share our results with you once the review is complete.  We have revised the sentence to ensure it is grammatically complete and reads more smoothly. The updated sentence now reads: 'Xulu-Gama and Hadebe argue for comprehensive language policies spanning all educational levels, including higher education, to enhance accessibility and inclusivity. | Page 3, Line 36 -38. |
| 3 | This paper is written in the future tense. Have you done this study, or are you planning to do this study? | Thank you for your comment. This paper presents a scoping review protocol, outlining the methodology and planned approach for the study. As such, it is written in the future tense to describe the steps that will be undertaken in the course of the review. |  |
|  | I should think that PLOS One would want to publish the results of your study. I also would like to see more citations to your research tools - such as the review will follow Arksey and O'Malley's framework and PRISMA-ScR guidelines. And JBI Manual for Evidence Synthesis. We have some info under methodology/scoping review about Arksey…. | Thank you for your comment. We have now ensured that all relevant citations are included. Specifically, we have referenced the Arksey and O'Malley framework and PRISMA-ScR guidelines, as well as the JBI Manual for Evidence Synthesis, which are fundamental to our methodology. | Page 5, Line 84 – 90. |
|  | citation? Rayyan software | We’ve cited Rayyan, as suggested. | Page 11, Line 165. |
|  | With regard to science education, the global language of science currently is English – not so much a concern for undergraduate education but a concern for graduate and post-graduate education, where publishing is required. | Thank you for your comment. We acknowledge that English is currently the global language of science, particularly in graduate and post-graduate education, where publishing and international collaboration are often essential. While this may not be as pressing at the undergraduate level, we believe that language policies should support linguistic diversity to ensure inclusivity and access to knowledge, while also recognizing the importance of English in academic publishing and research. |  |
|  | Population, Concept, Context framework - ?? | Regarding the Population, Concept, Context (PCC) framework, we have now included the relevant citation for this framework to clarify its inclusion in our study. | Page 6, line 98 – 99. |
| 4 | The study proposed is sound and valuable to the research community. However, the search strategy, which is the core component of data collection, could greatly benefit from additional breadth to ensure comprehensiveness. More synonyms are needed for each search topic, as are included in the attached document. Specifically, authors should plan to include the names of the universities that could be included in the study if possible, as well as, the specific languages that would be included. Use of both truncation (*) and proximity searching (NEAR/N) should also be considered. For example, "Language" NEAR/2 (policy or policies). Proximity searching is available syntactically in Web of Science, Scopus, and ERIC. | Thank you for the comment. We have expanded and refined our search strategy to enhance comprehensiveness, incorporating the recommended elements. Specifically, we have:  Broadened the search terms We now include multiple synonyms for key concepts such as "language policy," "language planning," and "language governance."  Explicitly included South African universities – the search string now contains the names of all major South African universities to capture institutional research.  Expanded language coverage – We have added official South African languages (e.g., isiZulu, Afrikaans, Sesotho) and other relevant languages  Applied advanced search techniques – where supported, Boolean operators (AND, OR), truncation (*), and proximity searching (NEAR/N) have been integrated.  Adapted search strategies for different databases:  Scopus, Web of Science, and ERIC: proximity operators (NEAR/N) and Boolean logic are used to refine searches.  Google Scholar: a simplified Boolean approach is applied, considering platform limitations.  African Journals Online (AJOL): the search is tailored to key terms, considering character limits and indexing constraints.  We believe these enhancements will strengthen the search strategy and ensure a more comprehensive review. The revised search strategy is detailed in the manuscript. | Page 7 - 10, Line 114 – 153. |
|  | Beyond the search, authors should consider eliminating the language filter for English described in the methodology. Although most studies are published in English, it seems counterproductive to exclude additional languages in a study about multilingual policies. | Thank you for your comment. We have eliminated the English-language filter from our search strategy to ensure inclusivity of relevant research. We will now include studies in additional languages and, where necessary, make use of translation services to accommodate non-English studies. All languages have been included in the search string. We believe this adjustment will enrich the scope and comprehensiveness of our review. | Page 8 – 10, line 137 – 153. |
| 5 | Although this scoping review protocol is quite well written, it is not suitable for publication in this journal, which accepts only systematic review and meta-analysis protocols. | Thank you for your comment. We appreciate your assessment of our scoping review protocol. |  |
